# Association of Pretreatment Physical and Geriatric Parameters with Treatment Tolerance and Survival in Elderly Patients with Stage I–II Non-Small Cell Lung Cancer: An Evaluation of Usual Care Data

**DOI:** 10.3390/cancers14235994

**Published:** 2022-12-05

**Authors:** Melissa J. J. Voorn, Merle F. R. Bootsma, Gerben P. Bootsma, Vivian E. M. van Kampen-van den Boogaart, Geerten J. A. van Riet, Dirk K. de Ruysscher, Bart C. Bongers, Maryska L. G. Janssen-Heijnen

**Affiliations:** 1 Department of Clinical Epidemiology, VieCuri Medical Center, 5912 BL Venlo, The Netherlands; 2 Adelante Rehabilitation Center, 5912 BL Venlo, The Netherlands; 3 Department of Epidemiology, GROW School for Oncology and Developmental Biology, Faculty of Health, Medicine and Life Sciences, Maastricht University, 6200 MD Maastricht, The Netherlands; 4 Department of Pulmonology, Zuyderland Medical Center, 6419 PC Heerlen, The Netherlands; 5 Department of Pulmonology, VieCuri Medical Center, 5912 BL Venlo, The Netherlands; 6 Department of Geriatrics, Zuyderland Medical Center, 6419 PC Heerlen, The Netherlands; 7 Department of Radiation Oncology (MAASTRO Clinic), GROW School for Oncology and Developmental Biology, Maastricht University Medical Center, 6200 MD Maastricht, The Netherlands; 8 Department of Nutrition and Movement Sciences, Nutrition and Translational Research in Metabolism (NUTRIM), Faculty of Health, Medicine and Life Sciences, Maastricht University, 6200 MD Maastricht, The Netherlands; 9 Department of Epidemiology, Care and Public Health Research Institute (CAPHRI), Faculty of Health, Medicine and Life Sciences, Maastricht University, 6200 MD Maastricht, The Netherlands

**Keywords:** lung cancer, pretreatment risk assessment, physical performance, geriatric assessment, treatment tolerance, prehabilitation

## Abstract

**Simple Summary:**

Non-small cell lung cancer is predominantly a disease of older people in whom treatment intolerance is common. To make well-informed shared decisions concerning treatment options, pretreatment screening and/or assessment might be useful to identify patients who are expected to benefit from preventive lifestyle interventions. These interventions aim to improve a patient’s physical fitness before and during cancer treatment, resulting in improved treatment tolerance and a reduction of posttreatment complications. In this study, different physical and geriatric parameters were associated with treatment intolerance and survival in patients ≥70 years with stage I–II NSCLC undergoing surgery or stereotactic ablative radiotherapy. Evaluation of pretreatment physical and geriatric performance seems highly recommended for shared decision-making and selection of patients who might benefit from preventive interventions before and/or during treatment.

**Abstract:**

In this study, the association of pretreatment physical and geriatric parameters with treatment tolerance and survival in elderly patients with stage I–II NSCLC was evaluated. Retrospective data for patients aged ≥70 years, diagnosed between 2016 and 2020 with stage I–II NSCLC, and who underwent surgery or stereotactic ablative radiotherapy (SABR) in a large Dutch teaching hospital were retrieved from medical records. Associations of pretreatment physical and geriatric parameters with treatment tolerance and survival were analyzed. Of 160 patients, 49 of 104 (47%) patients who underwent surgery and 21 of 56 (38%) patients who received SABR did not tolerate treatment. In univariable analysis, World Health Organization (WHO) performance status ≥ 2, short nutritional assessment questionnaire score > 1, short physical performance battery score ≤ 9, and geriatric-8 score ≤ 14 were significantly associated with postoperative complications. Forced expiratory volume of one second < 80% of predicted was significantly associated with intolerance of SABR. In multivariable analysis, WHO performance status ≥ 2 and diffusing capacity for carbon monoxide < 80% were significantly associated with decreased overall survival. This is the first study that investigated the association between pretreatment physical and geriatric parameters and treatment outcomes in patients with stage I–II NSCLC. Evaluation of physical and geriatric parameters before treatment initiation seems highly recommended to select patients who might benefit from preventive interventions before and/or during treatment.

## 1. Introduction

Lung cancer is the leading cause of cancer mortality worldwide [1]. It is predominantly a disease of older people, with half of all newly diagnosed patients being ≥70 years of age [1]. According to European guidelines [2], surgery is advised for relatively fit patients with resectable early-stage (stage I–II) non-small cell lung cancer (NSCLC). Stereotactic ablative radiotherapy (SABR) is the advised treatment for inoperable patients (e.g., due to a low physical fitness) and has shown similar survival rates [3]. Intensive treatment allows for longer disease-free and overall survival [3,4], but is often accompanied by treatment intolerance, such as no completion of treatment and/or unplanned hospitalizations [5]. In 2018, >35% of all operated patients with NSCLC had a postoperative complication, such as prolonged air leakage, bronchopneumonia, or bleeding. In patients undergoing SABR, 5–10% patients suffered from toxicity, such as dyspnea, pneumonitis, or lung fibrosis [6,7]. Patients with a higher risk for treatment complications are often characterized as aged ≥70 years, having tobacco-related comorbidity and/or cognitive impairment, being physically inactive and/or malnourished, and/or especially as having a low physiological reserve capacity (low aerobic fitness) [8,9].

In addition to making well-informed shared decisions concerning treatment options, pretreatment screening and/or assessment might be used to identify patients who are expected to benefit from pretreatment lifestyle interventions. These prehabilitation interventions aim to improve a patient’s physical fitness before and during cancer treatment. The comprehensive geriatric assessment (CGA) is a systematic procedure that objectively appraises the health status of elderly people, thereby focusing on somatic, functional, and psychosocial domains [10,11] and aiming to determine the presence of frailty in older people. Frailty is a loss of resources in several domains of functioning, which leads to a declined reserve capacity for dealing with psychophysiological stressors [12]. The CGA has historically been adopted to identify elderly patients who are unfit for intense oncologic treatment, but is time-consuming and therefore costly. Next to a geriatric assessment, specific physical function in older adults can be assessed by performance tests [13]. Timely identifying high-risk patients before the start of treatment is important to be able to initiate preventive interventions to improve treatment outcomes. It is still unclear to what extent these physical and geriatric tests are associated with treatment tolerance and survival in patients with NSCLC [14]. The aim of the present study was to gain insight into the association of pretreatment physical and geriatric parameters with treatment tolerance and survival in elderly patients with stage I–II NSCLC by evaluating usual care data.

## 2. Materials and Methods

### 2.1. Study Design and Patients

In this retrospective cohort study, real world usual care data from the medical records from Zuyderland, a large teaching hospital in the Netherlands, were used. This study started after approval of the Medical Research Ethics Committee Zuyderland (reference number: METCZ20200181). As a pretreatment physical and geriatric assessment is usual care for patients aged ≥70 years in Zuyderland, data of all patients aged ≥70 years who underwent curative intent treatment for stage I–II NSCLC (surgery or SABR) between 2016 and 2020 were included. Patients who underwent surgery or adjuvant chemotherapy for NSCLC in the year before diagnosis of the current tumor, patients who had radiotherapy to the ipsilateral thorax or mediastinum, patients with clinical superior vena cava syndrome, and patients who underwent previous cancer treatment within the last 3 years were excluded, because of the risk of biased outcomes.

### 2.2. Measurements

#### 2.2.1. Pretreatment Patient Characteristics

The following patient characteristics were obtained from the electronic patient files: age at diagnosis, sex (male, female), smoking status (current, former, never), lung cancer histology (adenocarcinoma, squamous cell carcinoma, large cell carcinoma/not otherwise specified), stage of disease (classified according to the clinical classification of the Tumor Node Metastases (cTNM) supplemented with the pathological TNM (8th edition of the TNM classification for non-small lung cancer) [15]), World Health Organization (WHO) performance status, adult comorbidity index-27 (ACE-27), body mass index (BMI), and the short nutritional assessment questionnaire (SNAQ). The WHO performance status was assessed by the case manager or pulmonologist to indicate the level of performance. Patients with a score ≥2 were classified as patients with a poor performance status [16]. Comorbidities were obtained using the ACE-27, a validated chart-based instrument. The ACE-27 grades specific conditions into levels of severity, grade 1 (mild), grade 2 (moderate), or grade 3 (severe). Based on the highest ranked single ailment, an overall comorbidity score (none to mild comorbidity (0 to 1) or moderate to severe comorbidity (≥2)) was assigned [10]. BMI was calculated as body mass divided by body height squared. BMI was categorized as underweight (<18.5 kg/m^2^) and normal and overweight (>18.5 kg/m^2^). Nutritional status was scored according to the SNAQ and subdivided into two categories: normal nutritional status (≤1) or malnourished (>1) [17].

#### 2.2.2. Pretreatment Physical Performance Parameters at Baseline

The following baseline physical performance parameters were obtained from the electronic patient files: forced expiratory volume in 1 second (FEV_1_), diffusing capacity for carbon monoxide (DLCO), short physical performance battery (SPPB), timed up-and-go (TUG) test, and handgrip strength (HGS). FEV_1_ and DLCO were both measured according to the ATS/ERS guideline [18] and expressed as a percentage of predicted based on sex and age [19]. Using spirometry, patients were asked to breathe in as deeply as possible, and then exhale as hard, quickly, and long as possible [18,20]. DLCO is a medical test that determines how much oxygen travels from the alveoli of the lungs to the blood stream [18]. Scores ≤ 80% of predictive for FEV_1_ and DLCO were classified as low [2]. The SPPB consists of (1) the ability to stand for up to 10 seconds with feet positioned in three ways (together side-by-side, semi-tandem, and tandem), (2) time to complete a 4-meter walk, and (3) time to rise from a chair five times without the hands resting on the armrests [21]. A total score < 9 points was indicated as having a lower level of functioning [21]. The TUG test measures of the duration required for the patient to rise from a chair, walk over a distance of 3 meters, turn around, walk back, and sit on the chair [22]. A score >12 seconds was indicated as having a lower level of functioning [22]. HGS is a reliable measure of maximum grip force evaluated using a handheld dynamometer (JAMAR Hydraulic Hand Dynamometer, JA Preston Corporation, Jackson, MI, USA) and was included as a measure of muscle strength. A value below the 10th percentile of the UK Biobank reference values, taking sex, age, and body height into account, in at least one side, was considered as handgrip weakness [23].

#### 2.2.3. Pretreatment Geriatric Assessment at Baseline

Based on the outcomes of a geriatric assessment and predefined cut-off points, patients were classified as fit or (pre)frail. The G8 screening tool consists of an 8-item questionnaire. It places significant weight on nutritional status (46% of the total score), but also focuses on functional mobility, neuropsychological problems, medication use, self-rated health status, and age [24]. Geriatric impairment was defined as a score ≤14 on the G8 screening tool [24]. The Groningen frailty indicator (GFI) is a short and easy to administer 15-item screening questionnaire to determine a person’s level of frailty [12]. The GFI screens for the loss of functions and resources in 4 domains of functioning: physical (functional mobility, multiple health problems, physical fatigue, vision, hearing), cognitive (cognitive functioning), social (emotional isolation), and psychological (depressed mood and feelings of anxiety). Geriatric impairment was defined as a score ≥4 on the GFI [12]. The definition of CGA vulnerability was based on previous research and defined as meeting the cut-off scores for impairment in two or more CGA domains [25,26], as an impairment in ≥2 domains has been found to increase the risk for future disability or mortality [27]. The following measurements were included in the CGA. Cognitive performance was measured by the Montreal cognitive assessment (MoCa) with a score <26 indicating cognitive impairment [28]. Depression was assessed with the hospital anxiety and depression scale (HADS) (>8 demonstrating at risk for depression) was used for psychological distress [29]. The instruments of Barthel and Katz were used to quantify the activities of daily living (ADL) (<10 indicating dependency) [16,30], the Lawton and Brody instrument for the instrumental activities of daily living (IADL) (<5 male/<8 female representing dependency) [31], history of falls (≥1), and the mini nutritional assessment (MNA) (<24 indicating at risk for malnutrition score) for nutritional status [32]. 

### 2.3. Outcomes of Treatment Tolerance and Survival

In case of surgery, treatment intolerance was defined as at least one of the following events occurring during a 30-day postoperative period: complications classified as Clavien-Dindo grade 2 or higher [33], at least one readmission, and/or a postoperative hospital length of stay > 5 days. In case of SABR, treatment intolerance was defined as toxicities grade 3 or higher according to the common terminology criteria for adverse events (CTCAE, v6.0) and/or at least one readmission. Overall survival (OS) was calculated as time from diagnosis of lung cancer until death from all causes.

### 2.4. Statistical Analyses

Data was analyzed using IBM SPPS Statistics for Windows version 24 (IBM Corp., Armonk, NY, USA). Descriptive statistics were used to summarize patient characteristics and cross-tabulations were used to analyze associations between pretreatment baseline patient and tumor characteristics, physical performance parameters, geriatric performance parameters, and type of treatment using chi^2^ tests for categorical variables and analysis of variance (ANOVA) for continuous variables. Associations of pretreatment baseline patient and tumor characteristics, physical performance parameters, and geriatric parameters with treatment intolerance were analyzed by univariable binary logistic regression analysis, according to treatment type. Because of small numbers, *p*-values < 0.10 were considered statistically significant. The odds ratios (ORs) and corresponding 90% confidence intervals (CIs) were displayed. An OR > 1.0 indicated poorer tolerance of treatment. Patients who were alive at the end of the study were censored. Univariable hazard ratios (HRs) and 90% CIs for associations of patient and tumor characteristics, physical parameters, geriatric parameters, and type of treatment with OS were calculated by Cox proportional hazards analyses. Because of small numbers, associations with a *p*-value <0.10 were considered statistically significant. Parameters with a *p*-value <0.10 in the univariable analyses were selected for the multivariable regression analyses. Worse survival compared to the reference group was indicated by a HR >1.0. 

## 3. Results

### 3.1. Pretreatment Patient Characteristics and Physical and Geriatric Parameters 

Data of 160 consecutive patients aged ≥70 years who were diagnosed with stage I–II NSCLC were included. An overview of patient and tumor characteristics according to type of treatment is presented in Table 1. Initial treatment consisted of surgery in 104 patients (65.0%) and SABR in 56 patients (35.0%). Stage I NSCLC was more common in patients receiving SABR (89.3%) compared to those undergoing surgery (60.6%). Patients receiving SABR had a statistically significant higher mean age (78.3 years) compared to patients undergoing surgery (75.7 years). Of the patients undergoing surgery, 58.7% had an adenocarcinoma and 41.3% had a squamous cell carcinoma, compared to 21.4% and 8.9% respectively for patients receiving SABR. In addition, stage I disease, WHO performance status ≥ 2, ACE-27 score ≥ 2, BMI < 18.5 kg/m^2^, SNAQ score > 1, FEV_1_ and DLCO < 80% of predicted, SPPB score ≤ 9, TUG test > 12 seconds, G8 ≤ 14, and GFI ≥ 4 were significantly more present among patients receiving SABR than among those undergoing surgery. 

A geriatric assessment was completed in 63.1% of the included patients. Geriatric assessment was omitted more often in patients undergoing SABR than in patients undergoing surgery. Patients who did not undergo a CGA more often had a large cell carcinoma/not otherwise specified and fewer readmissions. An overview of patient, tumor, and treatment characteristics in subgroups of fit patients, frail patients, and patients who did not undergo a CGA is shown in Table 2.

### 3.2. Treatment Intolerance 

A total of 70 patients (43.7%) did not tolerate treatment. Treatment intolerance occurred in 49 of 104 (47.1%) patients undergoing surgery and in 21 of 56 (37.7%) patients receiving SABR. Type of treatment intolerance, stratified for type of treatment, is shown in Table 3. In univariable regression analyses in patients undergoing surgery, stage II disease (OR 2.54), WHO performance status ≥2 (OR 4.46), SNAQ score > 1 (OR 2.84), SPPB score ≤ 9 (OR 4.14), G8 score ≤ 14 (OR 3.79), or a GFI score ≥ 4 (OR 3.40) were significantly associated with postoperative complications. An FEV_1_ < 80% of predicted (OR 5.33) was significantly associated with treatment intolerance in univariable regression analyses in patients receiving SABR. Results of the univariable regression analyses for intolerance of surgery respectively SABR are shown in Table 4. 

### 3.3. Overall Survival 

**Figure 1:**
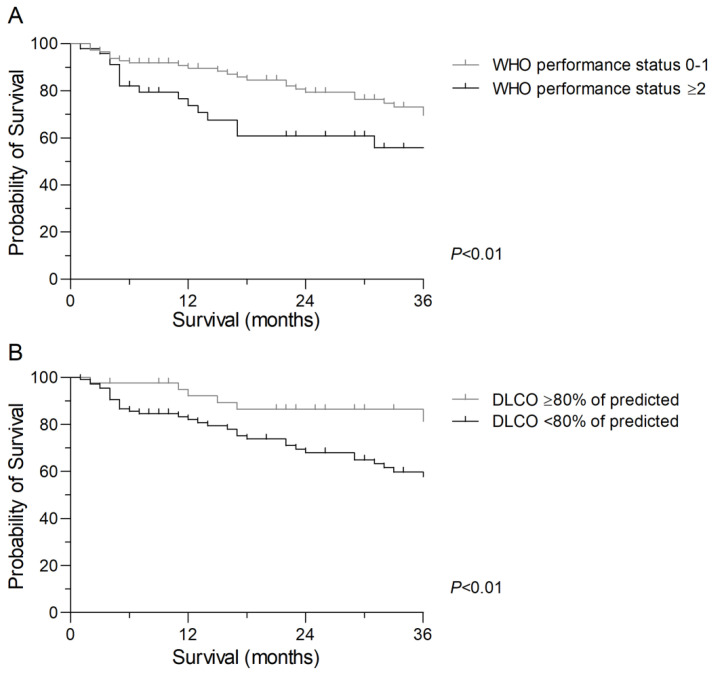
Kaplan–Meier survival curves for patients with non-small cell lung cancer who underwent surgery or stereotactic ablative radiotherapy. (**A**). Kaplan–Meier survival curve according to pretreatment World Health Organization (WHO) performance status (Log rank: *p* < 0.01). (**B**). Kaplan–Meier survival curve according to pretreatment diffusing capacity for carbon monoxide (DLCO) (Log rank: *p* < 0.01).

Median follow-up was 49 months. Median overall survival for the total group was 41 months, and at the time of analysis 50 patients (31.3%) had died. In univariable analyses, SABR (HR 2.00), squamous cell carcinoma or large cell carcinoma/not otherwise specified (HR 2.52 and 2.89), a WHO performance status ≥2 (HR 2.25, *p* < 0.01: Figure 1), a BMI < 18.5 kg/m^2^ (HR 2.69), a DLCO < 80% of predicted (HR 2.97, *p* < 0.01: Figure 1), a SPPB score ≤ 9 (HR 2.21), a TUG test > 12 seconds (HR 3.42), and treatment intolerance (HR 2.26) were significantly associated with poorer survival. The following factors were analyzed for their association with survival in multivariable analyses: type of treatment, histology, WHO performance status, and DLCO. Squamous cell carcinoma (HR 2.37), WHO performance status ≥ 2 (HR 2.03), and DLCO <80% of predicted (HR 2.37) remained significantly associated with poorer survival. Geriatric assessment variables were not included due to high proportions of missing values, whereas BMI was not included in multivariate analysis, because of a very low percentage of patients being underweight. Due to the high proportion of missing cases, the SPPB and TUG test were also excluded from multivariable analyses. Results of univariable and multivariable Cox regression analyses for survival are shown in Table 5.

## 4. Discussion

The aim of this study was to investigate associations of pretreatment physical and geriatric parameters with treatment tolerance and survival in patients aged ≥ 70 years with stage I–II NSCLC. Results demonstrated that several physical parameters and a limited number of pretreatment geriatric parameters were associated with treatment tolerance, with worse scores indicating a higher risk for adverse treatment outcomes. Moreover, worse performance on pretreatment physical parameters were significantly associated with reduced overall survival, whereas pretreatment geriatric parameters were not associated with survival.

In this study, patients with an FEV_1_ < 80% of predicted were more often selected for SABR, which is in line with European guidelines [2]. According to these guidelines [2], surgical risk is not increased when FEV_1_ and the DLCO are both ≥ 80% of predicted. Almost half (46%) of the patients with a WHO performance status ≥2 underwent SABR. Current study results and results of a previous study [34] therefore suggest that FEV_1_, DLCO, and WHO performance status have an added value in identifying patients at high risk for postoperative complications who are therefore advised to undergo SABR. However, even in patients with an adequate WHO performance status (0–1), outcome is heterogeneous [35], because geriatric impairments can still be present in patients with a WHO performance status of 0 or 1 (65.7%). Therefore, a more detailed evaluation of patient’s functional status may be of added value in addition to WHO performance status. 

Regarding physical parameters, only a SPPB score ≤ 9 and SNAQ score > 1 were associated with a higher risk for postoperative complications in this study, whereas a FEV_1_ <80% of predicted was related with a higher risk for intolerance of SABR. In addition to demonstrating that pretreatment screening of physical status is associated with both treatment intolerance and survival, information on the associations between physical status and recovery of physical functioning is also essential to make adequate treatment decisions together with patients. Also, specific pretreatment assessment of aerobic fitness using a cardiopulmonary exercise test (CPET) [36], steep ramp test (a short maximal test on a cycle ergometer that is strongly related to aerobic fitness) [37], or incremental shuttle walk test (iSWT) [38] with adequate cut-off points in patients with NSCLC might improve pretreatment risk assessment. A systematic review reported that a better performance on preoperative exercise tests, especially a higher aerobic fitness as objectively measured by the CPET, was associated with a lower risk for postoperative complications in patients with NSCLC [39]. Moreover, the iSWT and steep ramp test for estimating a patient’s preoperative aerobic fitness [37,38] might also be used to timely identify high-risk patients who might benefit from lifestyle interventions (e.g., physical exercise training) before and during cancer treatment (prehabilitation and early rehabilitation, respectively) [40].

In the current univariable analyses, physical parameters were associated with poorer survival in patients undergoing surgery or SABR. This agrees with a previous study in patients with lung cancer [41]. The association between physical parameters and survival might partly be explained by the fact that patients with a poor physical status suffered more often from treatment intolerance. This means that especially patients with a poor physical status could benefit from pretreatment preventive lifestyle interventions. Physical exercise training on top of medical treatment could optimize physical status, leading to better tolerance of intensive treatment [42] and preservation of physical functioning. This can be achieved by exercise prehabilitation (physical exercise training before treatment initiation). The physiological reserve capacity can be increased by a combination of aerobic and resistance training [42]. Even better outcomes might be achieved when the diet is adapted to the needs of training as well, including healthy and protein-rich products [43]. The univariable analysis also showed that patients receiving SABR had a significantly worse survival than patients undergoing surgery. However, this association disappeared after adjusting for differences in baseline characteristics between patients undergoing surgery and patients receiving SABR. This is in line with previous research demonstrating that outcomes between SABR and surgery for operable patients with stage I NSCLC are comparable [5]. For shared decision-making, it is therefore important to gain insight into patient characteristics that are associated with the risks and benefits of both treatment options [5].

With respect to pretreatment geriatric parameters, a frailty score determined from the geriatric screening tools G8 or GFI was associated with complications after surgery, but not with intolerance of SABR. The latter is in line with previous research in patients with head and neck cancer undergoing radiotherapy [44]. It is likely that the gradual increase in complaints during radiation treatment in vulnerable patients is better tolerated than the major impact of the surgery-induced stress response. As frailty refers to decreases in physiological reserves after a stressful event [45], one can speculate that the duration and intensity of the stress response are an important aspect. In contrast, when the stress response is prolonged and less intense, which is the case with radiation therapy, the patient can adapt to disrupted homeostasis. Although not supported by the current study findings, a geriatric assessment is able to detect unidentified but manageable problems [46]. Therefore, a geriatric screening might lead to better outcomes using targeted prehabilitation interventions to improve treatment tolerance and by adjusting oncologic treatment plans in the elderly cancer population [46].

Despite the novelty of prognostic physical and geriatric parameters in patients with NSCLC aged > 70 years and undergoing surgery or SABR, results reported in this study need to be interpreted with caution due to some limitations. In the current retrospective observational study, a geriatric assessment was not performed in 36.9% of the patients. To provide a good overview of usual care data, it was decided to present all data and to also provide insight into the group without pretreatment geriatric assessments. Due to the large proportion of missing data, information from detailed geriatric and physical parameters could unfortunately not be included in the multivariable regression analyses. This might have biased the results, since the group of patients in whom no geriatric assessment had been performed more often received SABR, more often had a large cell carcinoma/not otherwise specified, and had fewer readmissions. Failure to refer a patient for a pretreatment geriatric assessment might be explained by the fact that SABR has become the standard of care for medically inoperable early-stage NSCLC [47], regardless of poor WHO performance status or physical status. However, both the International Society for Geriatric Oncology and the National Comprehensive Cancer Network recommend that elderly patients with cancer undergo a geriatric assessment prior to treatment decisions to detect problems which may not promptly be identified by routine physical examinations or medical history. This geriatric assessment can be used to predict treatment intolerance and survival, and to support treatment decisions [48]. Furthermore, only patients who were already selected for surgery or SABR were included in this study. This means that results were predominantly based on relatively fit patients. Therefore, caution is warranted when extrapolating the current results.

A worse physical and geriatric status is often associated with treatment intolerance and worse survival in patients with cancer, especially in those undergoing surgery [49]. However, uncertainty remains in this study about the discriminative power of the used physical and geriatric screening and assessment tools for selecting patients for the right treatment and to discuss the risks and benefits of the treatment with the patient. According to the current study results and results from a previous study [39], it appears to be useful to use pretreatment physical performance tests for assessing physical fitness (e.g., aerobic fitness, functional mobility) to select patients who might benefit from preventive interventions before and during treatment. For future research, it is recommended to conduct a large prospective multicenter study in which a large group of patients aged ≥70 years of age perform easy-to-use physical exercise tests and geriatric assessments before treatment initiation to clarify which (combination of) pretreatment parameters are predictive for treatment tolerance and survival. This may contribute to the development of a multimodal tool for pretreatment risk assessment.

## 5. Conclusions

Several physical and geriatric parameters were associated with treatment tolerance and survival in patients aged ≥ 70 years with stage I–II NSCLC undergoing surgery or SABR, in which worse scores indicate a higher risk for adverse treatment outcomes. An evaluation of pretreatment physical and geriatric performance seems highly recommended for shared decision-making and selecting patients who might benefit from preventive interventions before and/or during treatment. Further research is needed, particularly in patients receiving SABR, to investigate the ability of pretreatment physical exercise tests and geriatric assessments to accurately identify patients with stage I–II NSCLC who have an increased risk for treatment intolerance, as these patients might benefit from prehabilitation interventions to improve their physical performance status before treatment initiation. 

## Figures and Tables

**Table 1:** Overview of patient and tumor characteristics (including pretreatment physical and geriatric parameters at baseline) of patients with stage I–II NSCLC aged ≥70 years according to treatment modality.

Parameters	Surgery n = 104 n (%)	SABR n = 56 n (%)	*p*-Value
**Mean ± SD age (years)**	75.7 ± 4.3	78.3 ± 5.2	**<0.01**
Sex			
Male	61 (58.7)	32 (57.1)	0.85
Female	43 (41.3)	24 (42.9)	
Smoking status			
Current	36 (34.6)	20 (35.7)	
Former	52 (50.0)	30 (53.6)	0.61
Never	15 (14.4)	5 (8.9)	
Lung cancer histology			
Adenocarcinoma	61 (58.7)	12 (21.4)	
Squamous cell carcinoma	43 (41.3)	5 (8.9)	**<0.01**
Large cell carcinoma/not otherwise specified	0 (0.0)	39 (69.6)	
Stage of disease			
Stage I	63 (60.6)	50 (89.3)	**<0.01**
Stage II	41 (39.4)	6 (10.7)	
WHO performance status			
0–1	83 (79.8)	30 (53.6)	
≥2	17 (16.3)	26 (46.4)	**<0.01**
Unknown	4 (3.8)	0 (0.0)	
ACE-27			
0–1	77 (74.8)	32 (58.2)	**0.03**
≥2	26 (25.2)	23 (41.8)	
BMI			
Normal and overweight (>18.5 kg/m^2^)	101 (97.1)	50 (89.3)	
Underweight (<18.5 kg/m^2^)	3 (2.9)	5 (8.9)	**0.09**
Unknown ^a^	0 (0.0)	1 (1.8)	
SNAQ score			
Adequate nutritional status (≤1)	87 (83.7)	39 (69.6)	**0.04**
Malnourished (>1)	16 (15.4)	16 (28.6)	
**Pretreatment physical parameters**			
FEV_1_			
≥80% of predicted	57 (54.8)	16 (28.6)	
<80% of predicted	47 (45.2)	37 (66.1)	**<0.01**
Unknown ^a^	0 (0.0)	3 (5.4)	
DLCO			
≥80% of predicted	36 (34.6)	6 (10.7)	
<80% of predicted	62 (59.6)	46 (82.1)	**<0.01**
Unknown ^a^	6 (5.8)	4 (7.1)	
SPPB score			
Higher level of functioning (>9)	25 (24.0)	8 (14.3)	
Lower level of functioning (≤9)	47 (45.2)	17 (30.4)	**<0.01**
Not assessed ^b^	32 (30.8)	31 (55.4)	
TUG test			
Higher level of functioning (≤12 s)	56 (53.8)	16 (28.6)	
Lower level of functioning (>12 s)	5 (4.8)	5 (8.9)	**0.06**
Not assessed ^b^	43 (41.3)	35 (62.5)	
Handgrip strength ^c^			
Normal	48 (46.2)	17 (30.4)	
Weak	5 (4.8)	2 (3.6)	0.89
Not assessed ^b^	51 (49.0)	37 (66.1)	
**Pretreatment geriatric parameters**			
G8			
Fit (>14)	31 (29.8)	6 (10.7)	
Frail (≤14)	37 (35.6)	19 (33.9)	**0.06**
Not assessed ^b^	36 (34.6)	31 (55.4)	
GFI			
Fit (<4)	43 (41.3)	9 (16.1)	
Frail (≥4)	29 (27.9)	18 (32.1)	**0.02**
Not assessed ^b^	32 (30.8)	29 (51.8)	
**Pretreatment comprehensive geriatric assessment**			
CGA			
Fit (<2)	24 (23.1)	9 (16.1)	
Frail (≥2)	49 (47.1)	19 (33.9)	0.94
Not assessed ^b^	31 (29.8)	28 (50.0)	
MoCa ^d^			
Fit (≥26)	27 (26.0)	11 (19.6)	0.83
Frail (<26)	46 (44.2)	17 (30.4)	
HADS depression ^d^			
No risk at depression (≤8)	66 (63.5)	25 (44.6)	0.87
Risk at depression (>8)	7 (6.7)	3 (5.4)	
Barthel and Katz ADL ^d^			
No restrictions (≥10)	11 (10.6)	4 (7.1)	0.92
Restrictions (<10)	62 (59.6)	24 (42.9)	
Lawton and Brody IADL ^d^			
No restrictions (≥5 male, ≥8 female)	54 (51.9)	19 (33.9)	0.54
Restrictions (<5 male, <8 female)	19 (18.3)	9 (16.1)	
History of falls ^d^			
<1	65 (62.5)	19 (33.9)	**0.01**
≥1	8 (7.7)	9 (16.1)	
MNA ^d^			
Normal nutritional status (≤1)	1 (1.0)	2 (3.6)	0.13
Malnourished (>1)	72 (69.2)	26 (46.4)	

Data are presented as means ± SD or n (%). Bold *p*-values indicate statistical significance. Abbreviations: ACE-27 = adult comorbidity index-27; ADL = activities of daily living; BMI = body mass index; CGA = comprehensive geriatric assessment; DLCO = diffusing capacity for carbon monoxide; FEV1 = forced expiratory volume in 1 second; GFI = Groningen frailty index; HADS = hospital anxiety and depression scale; IADL = instrumental activities of daily living; MNA = mini nutritional assessment; MoCa = Montreal cognitive assessment; SABR = stereotactic ablative radiotherapy; SD = standard deviation; SNAQ = short nutritional assessment questionnaire; SPPB = short physical performance battery; TUG = timed up-and-go; WHO = World Health Organization. ^a^: unknown represents missing data and was not included in statistical analyses. ^b^: patients that were not assessed were not included in the statistical analyses. ^c^: a score below the 10th percentile of norm values [23]. ^d^: the number and percentages of patients that were ‘not assessed’ are similar as for ‘CGA’.

**Table 2:** Overview of patient, tumor, and treatment characteristics in relation to the pretreatment comprehensive geriatric assessment.

Pretreatment Comprehensive Geriatric Assessment				
Parameters	Fit (n = 33) n (%)	Frail (n = 68) n (%)	Not Assessed (n = 59) n (%)	*p*-Value ^a^
Mean ± SD age (years)	75.2 ± 4.9	77.4 ± 5.1	76.5 ± 4.2	**0.08**
Sex				
Male	21 (63.6)	40 (58.8)	32 (54.2)	0.67
Female	12 (36.4)	28 (41.2)	27 (45.8)	
Smoking status				
Current	15 (46.9)	23 (34.3)	10 (16.9)	
Former	15 (46.9)	36 (53.7)	31 (52.5)	0.41
Never	2 (6.3)	8 (11.9)	18 (30.5)	
Lung cancer histology				
Adenocarcinoma	13 (39.4)	31 (45.6)	29 (49.2)	
Squamous cell carcinoma	12 (36.4)	26 (38.2)	10 (16.9)	**0.04**
Large cell carcinoma/not otherwise specified	8 (24.2)	11 (16.2)	20 (33.9)	
Stage of disease				
Stage I	26 (78.8)	37 (54.4)	50 (84.7)	**<0.01**
Stage II	7 (21.2)	31 (45.6)	9 (15.3)	
Type of treatment				
Surgery	24 (72.7)	49 (72.1)	31 (52.5)	**0.04**
SABR	9 (27.3)	19 (27.9)	28 (47.5)	
WHO performance status				
0–1	24 (72.7)	44 (65.7)	45 (80.4)	
≥2	9 (27.3)	23 (34.3)	11 (19.6)	0.19
Unknown	0	1	3	
ACE-27				
0–1	24 (72.7)	49 (72.1)	36 (63.2)	0.49
≥2	9 (27.3)	19 (27.9)	21 (36.8)	
BMI				
Normal and overweight (≥18.5 kg/m^2^)	33 (100.0)	65 (95.6)	53 (91.4)	
Underweight (<18.5 kg/m^2^)	0 (0.0)	3 (4.4)	5 (8.6)	0.19
Unknown	0	0	1	
SNAQ score				
Adequate nutritional status (≤1)	23 (71.9)	56 (83.6)	47 (79.7)	
Malnourished (>1)	9 (28.1)	11 (16.4)	12 (20.3)	0.40
Unknown	1	1	0	
**Pretreatment physical parameters**				
FEV_1_				
≥80% of predicted	18 (54.5)	28 (41.8)	27 (47.4)	
<80% of predicted	15 (45.5)	39 (58.2)	30 (52.6)	0.48
Unknown	0	1	2	
DLCO				
≥80% of predicted	6 (18.8)	19 (29.7)	17 (31.5)	
<80% of predicted	26 (81.3)	45 (70.3)	37 (68.5)	0.41
Unknown	1	4	5	
**Treatment intolerance**				
Clavien-Dindo grade ≥ 2 or CTCAE grade ≥ 3	11 (33.3)	26 (38.8)	14 (23.7)	0.19
Readmission	11 (33.3)	23 (33.8)	9 (15.5)	**<0.05**
Postoperative hospital length of stay >5 days	13 (54.2)	27 (55.1)	11 (37.9)	0.31
Survival				
1–year	84.8	83.8	89.8	0.60
3–year	69.7	73.5	81.4	0.40

Data are presented as means ± SD or n (%). Bold *p*-values indicate statistical significance. Abbreviations: ACE-27 = adult comorbidity index-27; BMI = body mass index; CTCAE = common terminology criteria for adverse events; DLCO = diffusing capacity for carbon monoxide; FEV_1_ = forced expiratory volume in 1 second; MoCa = Montreal cognitive assessment; SABR = stereotactic ablative radiotherapy; SD = standard deviation; SNAQ = short nutritional assessment questionnaire; TUG = timed up-and-go; WHO = World Health Organization. ^a^: unknown represents missing data and was not included in statistical analyses.

**Table 3:** Type of treatment intolerance, stratified for type of treatment.

Clavien-Dindo Classification	Surgery (n = 104) n (%)	CTCAE Grade	SABR (n = 56) n (%)
0–I	58 (55.8)	0–II	48 (85.7)
II	28 (26.9)	III	4 (7.1)
III	9 (8.7)	IV	1 (1.8)
IV	4 (3.8)	V	1 (1.8)
V	5 (4.8)		
No readmission	79 (76.0)	No readmission	38 (67.9)
Readmission	25 (24.0)	Readmission	18 (32.1)

Abbreviations: CTCAE = common terminology criteria for adverse events; SABR = stereotactic ablative radiotherapy.

**Table 4:** Univariable odds ratios for associations of pretreatment patient characteristics, physical parameters, and geriatric parameters with intolerance of treatment in patients with stage I–II NSCLC, stratified for type of treatment.

	Surgery (n = 104) Treatment Intolerance n = 49 (47%)		SABR (n = 56) Treatment Intolerance n = 21 (38%)	
	Univariable		Univariable	
	OR (90% CI)	*p*-Value	OR (90% CI)	*p*-Value
Age (continuous, in years)	1.01 (0.93–1.11)	0.78	0.96 (0.86–1.07)	0.46
Sex				
Male	Reference		Reference	
Female	1.82 (0.83–4.00)	0.14	1.00 (0.34–2.98)	1.00
Smoking status				
Current	Reference		Reference	
Former	0.60 (0.18–2.03)	0.41	0.82 (0.11–5.99)	0.84
Never	0.53 (0.16–1.70)	0.29	0.76 (0.11–5.24)	0.78
Stage of disease				
Stage I	Reference		NI ^a^	
Stage II	**2.54 (1.13** **–5.69)**	**0.02**		
WHO performance status				
0–1	Reference		Reference	
≥2	**4.46 (1.34** **–14.83)**	**0.02**	1.47 (0.49–4.35)	0.49
ACE–27				
0–1	Reference		Reference	
≥2	1.14 (0.47–2.77)	0.77	1.23 (0.40–3.73)	0.71
BMI				
Normal and overweight (≥18.5 kg/m^2^)	NI ^a^		NI ^a^	
Underweight (<18.5 kg/m^2^)				
SNAQ score				
Adequate nutritional status (≤1)	Reference		Reference	
Malnourished (>1)	**2.84 (0.91** **–8.86)**	**0.07**	1.56 (0.47–5.12)	0.47
**Pretreatment physical parameters**				
FEV_1_				
≥80% of predicted	Reference		Reference	
<80% of predicted	0.84 (0.39–1.82)	0.65	**5.33 (1.06–26.90)**	**0.04**
DLCO				
≥80% of predicted	Reference		NI ^a^	
<80% of predicted	1.89 (0.81–4.38)	0.14		
SPPB				
Higher level of functioning (>9)	Reference		Reference	
Lower level of functioning (≤9)	**4.14 (1.45–11.87)**	**0.01**	2.38 (0.42–13.39)	0.33
TUG test				
Higher level of functioning (≤12 s)	NI ^a^		Reference	
Lower level of functioning (>12 s)			0.52 (0.07–4.00)	0.52
Handgrip strength				
Normal	NI ^a^		NI ^a^	
Weak ^b^				
**Pretreatment geriatric parameters**				
G8				
Fit (>14)	Reference		Reference	
Frail (≤14)	**3.79 (1.38–10.37)**	**0.01**	0.36 (0.05–2.50)	0.30
GFI				
Fit (<4)	Reference		Reference	
Frail (≥4)	**3.40 (1.26–9.21)**	**0.02**	0.32 (0.06–1.71)	0.18
**Pretreatment comprehensive geriatric assessment**				
CGA				
Fit (<2)	Reference		Reference	
Frail (≥2)	1.04 (0.39–2.77)	0.94	0.51 (0.12–2.88)	0.50
MoCa				
Fit (≥26)	Reference		Reference	
Frail (<26)	0.73 (0.28–1.91)	0.52	0.31 (0.06–1.51)	0.15
HADS depression				
No risk for depression (≤8)	NI ^a^		NI ^a^	
Risk for depression (>8)				
Barthel and Katz ADL				
No restrictions (≥10)	Reference		Reference	
Restrictions <10	0.54 (0.14–2.02)	0.36	0.85 (0.10–7.04)	0.88
Lawton and Brody IADL				
No restrictions (≥5 male, ≥8 female)	Reference		Reference	
Restrictions (<5 male, <8 female)	0.84 (0.29–2.38)	0.74	0.89 (0.18–4.38)	0.89
History of falls				
<1	NI ^a^		3.43 (0.65–18.22)	0.15
≥1				
MNA				
Normal nutritional status (≤1)	NI ^a^		Reference	
Malnourished (>1)			0.86 (0.05–15.22)	0.92

Data are presented as means ± SD or n (%). Bold values indicate a statistically significant poorer tolerance of treatment. Abbreviations: ACE-27 = adult comorbidity index-27; ADL = activities of daily living; BMI = body mass index; CGA = comprehensive geriatric assessment; CI = confidence interval; DLCO = diffusing capacity for carbon monoxide; FEV_1_ = forced expiratory volume in 1 second; GFI = Groningen frailty index; HADS = hospital anxiety and depression scale; IADL = instrumental activities of daily living; MNA = mini nutritional assessment; MoCa = Montreal cognitive assessment; NI = not included; NSCLC = non-small cell lung cancer; OR = odds ratio; SABR = stereotactic ablative radiotherapy; SD = standard deviation; SNAQ = short nutritional assessment questionnaire; SPPB = short physical performance battery; TUG = timed up-and-go; WHO = World Health Organization. ^a^: not included in statistical analyses because the numbers in subgroups were too small. ^b^: a score below the 10th percentile of norm values [23].

**Table 5:** Univariable and multivariable hazard ratios and 95% CIs for associations of pretreatment patient, tumor, and treatment characteristics with overall survival in patients with stage I–II NSCLC.

	1-Year Survival %	3-Year Survival %	Univariable		Multivariable	
			HR (90% CI)	*p*-Value	HR (90% CI)	*p*-Value
Age	-	-	0.97 (0.92–1.03)	0.37	NI ^a^	
Type of treatment						
Surgery	88.5	79.8	Reference		Reference	
SABR	82.1	67.9	**2.00 (1.15** **–3.51)**	**0.01**	**1.73 (0.74** **–4.07)**	0.29
Sex						
Male	83.9	72.0	Reference		NI ^a^	
Female	89.6	80.6	0.68 (0.38–1.21)	0.19		
Smoking status						
Current	83.3%	75.0%	Reference			
Former	78.8%	63.3%	1.26 (0.66–2.43)	0.49	NI ^a^	
Never	73.3%	60.0%	1.85 (0.82–4.18)	0.14		
Histology						
Adenocarcinoma	95.9	83.6	Reference		Reference	
Squamous cell carcinoma	75.0	70.8	**2.52 (1.25** **–5.01)**	**0.01**	**2.37 (1.31** **–4.27)**	**0.02**
Large cell carcinoma/not otherwise specified	82.1	66.7	**2.89 (1.44** **–5.82)**	**<0.01**	1.53 (0.80–2.92)	0.28
Stage of disease						
Stage I	88.5	77.9	Reference		NI ^a^	
Stage II	80.9	70.2	1.36 (0.77–2.41)	0.29		
WHO performance status						
0-1	90.3	79.6	Reference		Reference	
≥2	74.4	62.8	**2.25 (1.24** **–4.10)**	**<0.01**	**2.03 (1.16** **–3.53)**	**0.04**
ACE-27						
0-1	87.2	75.2	Reference		NI ^a^	
≥2	85.7	77.6	1.08 (0.56–2.09)	0.81		
BMI						
Normal weight (≥18.5 kg/m^2^)	87.4	76.8	Reference		NI ^b^	
Underweight (<18.5 kg/m^2^)	75.0	62.5	**2.69 (0.96** **–7.59)**	**0.06**		
SNAQ score						
Adequate nutritional status (≤1)	85.7	76.2	Reference		NI ^a^	
Malnourished (>1)	87.5	75.0	1.46 (0.76–2.81)	0.262		
**Pretreatment physical parameters**						
FEV_1_						
≥80% of predicted	87.7	78.1	Reference		NI ^a^	
<80% of predicted	85.7	73.8	1.35 (0.77–2.40)	0.31		
DLCO						
≥80% of predicted	92.9	88.1	Reference		Reference	
<80% of predicted	83.3	69.4	**2.97 (1.33** **–6.62)**	**<0.01**	**2.37 (1.17** **–4.77)**	**0.04**
SPPB						
Higher level of functioning (>9)	92.7	80.0	Reference		NI ^c^	
Lower level of functioning (≤9)	73.8	61.9	**2.21 (1.14** **–4.26)**	**0.02**		
TUG test						
Higher level of functioning (≤12 s)	88.9	75.0	Reference		NI ^c^	
Lower level of functioning (>12 s)	50.0	30.0	**3.42 (1.52** **–7.70)**	**<0.01**		
Handgrip strength						
Normal	84.6	75.4	Reference		NI ^a^	
Weak ^d^	71.4	57.1	2.30 (0.78–6.77)	0.13		
**Pretreatment geriatric parameters**						
G8						
Fit (>14)	89.2	78.4	Reference		NI ^a^	
Frail (≤14)	78.6	67.9	1.60 (0.81–3.16)	0.18		
GFI						
Fit (<4)	86.5	72.1	Reference		NI ^a^	
Frail (≥4)	80.9	74.5	1.11 (0.57–2.22)	0.76		
**Pretreatment comprehensive geriatric assessment**						
CGA						
Fit (<2)	84.8	69.7	Reference		NI ^a^	
Frail (≥2)	83.8	73.5	0.77 (0.38–1.53)	0.45		
MoCa						
Fit (≥26)	84.2	71.1	Reference		NI ^a^	
Frail (<26)	84.1	73.0	0.72 (0.38–1.39)	0.33		
HADS depression						
No risk at depression (≤8)	84.6	71.4	Reference		NI ^a^	
Risk at depression (>8)	80.0	80.0	0.59 (0.14–2.46)	0.47		
Barthel and Katz ADL						
Fit (≥10)	73.3	60.0	Reference		NI ^a^	
Frail (<10)	86.0	74.4	0.77 (0.34–1.75)	0.53		
Lawton and Brody IADL						
Fit (≥5 male, ≥8 female)	83.6	72.6	Reference		NI ^a^	
Frail (<5 male, <8 female)	85.7	71.4	0.86 (0.43–1.71)	0.67		
History of falls						
<1	86.9	73.8	Reference		NI ^a^	
≥1	70.6	64.7	1.67 (0.79–3.54)	0.12		
MNA						
Adequate nutritional status (≤1)	100.0	100.0	Reference		NI ^a^	
Malnourished (>1)	83.7	71.4	0.90 (0.12–6.59)	0.92		
**Treatment intolerance**						
Treatment intolerance						
No	95.5	84.3	Reference		NI	
Yes	74.6	64.8	**2.26 (1.27** **–4.03)**	**<0.01**		

Bold values indicate a statistically significant worse survival. Abbreviations: ACE-27 = adult comorbidity index-27; ADL = activities of daily living; BMI = body mass index; CGA = comprehensive geriatric assessment; CI = confidence interval; DLCO = diffusing capacity for carbon monoxide; FEV_1_ = forced expiratory volume in 1 second; GFI = Groningen frailty index; HADS = hospital anxiety and depression scale; HR = hazard ratio; IADL = instrumental activities of daily living; MNA = mini nutritional assessment; MoCa = Montreal cognitive assessment; NSCLC = non-small cell lung cancer; SABR = stereotactic ablative radiotherapy; SNAQ = short nutritional assessment questionnaire; SPPB = short physical performance battery; TUG = timed up-and-go; WHO = World Health Organization. ^a^: not included when *p*-value ≥ 0.10. ^b^: BMI was not included in multivariate analysis, because of a low percentage of patients being underweight (5%). ^c^: SPPB and TUG test were not included in multivariate analysis, because of a high percentage of missing cases (39% and 49%) and because of violating the proportional hazards assumption. ^d^: a score below the 10th percentile of norm values [23].

## Data Availability

The data that support the findings of this study are available from Zuyderland Medical Center, but restrictions apply to the availability of these data. The data have been used under license for the current study and are therefore not publicly available. Data might however become available from the authors upon reasonable request and only after obtained permission from Zuyderland Medical Center.
